# 'Be active, eat right', evaluation of an overweight prevention protocol among 5-year-old children: design of a cluster randomised controlled trial

**DOI:** 10.1186/1471-2458-9-177

**Published:** 2009-06-08

**Authors:** Lydian Veldhuis, Mirjam K Struijk, Willemieke Kroeze, Anke Oenema, Carry M Renders, Anneke MW Bulk-Bunschoten, Remy A HiraSing, Hein Raat

**Affiliations:** 1 Department of Public Health, Erasmus MC University Medical Center Rotterdam, PO Box 2040, 3000 CA, Rotterdam, the Netherlands; 2 Institute of Health Sciences, Faculty of Earth and Life Sciences, VU University, Amsterdam, the Netherlands; 3 Department of Public and Occupational Health, Institute for Research in Extramural Medicine, VU Medical Center, Van der Boechorstraat 7, 1081 BT, Amsterdam, the Netherlands

## Abstract

**Background:**

The prevalence of overweight and obesity in children has at least doubled in the past 25 years with a major impact on health. In 2005 a prevention protocol was developed applicable within Youth Health Care. This study aims to assess the effects of this protocol on prevalence of overweight and health behaviour among children.

**Methods and design:**

A cluster randomised controlled trial is conducted among 5-year-old children included by 44 Youth Health Care teams randomised within 9 Municipal Health Services. The teams are randomly allocated to the intervention or control group. The teams measure the weight and height of all children. When a child in the intervention group is detected with overweight according to the international age and gender specific cut-off points of BMI, the prevention protocol is applied. According to this protocol parents of overweight children are invited for up to three counselling sessions during which they receive personal advice about a healthy lifestyle, and are motivated for and assisted in behavioural change.

The primary outcome measures are Body Mass Index and waist circumference of the children. Parents will complete questionnaires to assess secondary outcome measures: levels of overweight inducing/reducing behaviours (i.e. being physically active, having breakfast, drinking sweet beverages and watching television/playing computer games), parenting styles, parenting practices, and attitudes of parents regarding these behaviours, health-related quality of life of the children, and possible negative side effects of the prevention protocol. Data will be collected at baseline (when the children are aged 5 years), and after 12 and 24 months of follow-up. Additionally, a process and a cost-effectiveness evaluation will be conducted.

**Discussion:**

In this study called 'Be active, eat right' we evaluate an overweight prevention protocol for use in the setting of Youth Health Care. It is hypothesized that the use of this protocol will result in a healthier lifestyle of the children and an improved BMI and waist circumference.

**Trial registration:**

Current Controlled Trials ISRCTN04965410

## Background

### Childhood overweight and obesity

The prevalence of overweight and obesity among children has at least doubled over the past 25 years, especially in socially disadvantaged and specific ethnic subgroups [1-6]. In the Netherlands, in 2003 the prevalence of overweight (obesity included) among boys and girls aged about 5 years was 12.8% and 17.5%, respectively, compared with 5.2% and 8.6%, respectively, in 1980 [7].

Adverse health effects of obesity in children are: type 2 diabetes, hypertension, high cholesterol levels, apnoea during sleep, psychosocial problems and a lower quality of life [8-12]. Being overweight or obese as a child increases the risk of becoming an overweight or obese adult [13], and is associated with increased morbidity and mortality [13-17]. Therefore, prevention of childhood overweight and obesity is important. To prevent and curtail the increase of overweight and obesity in children, evidence-based prevention programs are needed.

### Preventing childhood overweight and obesity

Studies have suggested that the methods for prevention of overweight and obesity in childhood are family-based intervention programs that include personal advice about a healthy lifestyle and counselling behavioural changes. Such programs should focus on a combination of inducing healthy nutritional behaviour (i.e. having family breakfast daily and reducing intake of sweet beverages) and reducing sedentary behaviour (i.e. inducing being physically active and reducing watching TV/playing computer games) [18-21]. The parents' role is of particular importance for the behaviour of children, especially among young children. Parents directly determine the physical and social environment of children, and indirectly influence behaviour and habits through socialization processes and modelling [22,23]. It is also recommended that more attention should be given to long-term sustainability and incorporating of interventions in daily practice [20].

The Netherlands has a unique system for the maintenance of the health of children, i.e. the Youth Health Care (YHC) system. All children (0–19 years) are monitored by a nation-wide program at set ages. This program is offered free of charge by the government; participation is voluntary. The attendance rate is 95%. During the YHC check-ups the growth of each child is measured [24]. In 2005 a consensus-based protocol was developed to be applied in the YHC setting for the prevention of overweight and obesity in children aged 0 to 19 years [25]. The Municipal Health Services (MHSs) are preparing the implementation of this prevention protocol; however, before wide-scale implementation an effect evaluation of the protocol is needed.

### Objectives

The first YHC check-up during school age is at 5–6 years: an important moment to consider the prevention of overweight. The aim of the study 'Be active, eat right' is to assess the effectiveness of the prevention protocol among children with overweight. The design of the study is described below.

#### The study hypotheses

The hypotheses of the study are that, after two years of follow-up, compared with the control group the overweight children in the intervention group will:

- have reduced BMI and waist circumference

- more frequently have family breakfast on a daily basis, and consume less sweet beverages

- spend more time being physically active and less time watching television/playing computer games

We apply a cluster design with YHC teams (physician, nurse and assistant) as the unit of randomisation. Randomisation at the individual level (i.e. the level of the children) may lead to contamination of the control group [26]. The outcome measures of the study (BMI, waist circumference, and levels of inducing/reducing overweight behaviours) are performed at the individual level. The follow-up measures will be compared between the intervention and control group, taking into account the baseline values.

## Methods and design

### Study design

This cluster randomised controlled trial is conducted in the Netherlands among children aged about 5 years and their parents, who are invited by the MHSs for a regular preventive health check. The YHC teams that perform the check consist of a physician, a nurse and an assistant; they form the unit of randomisation. The randomisation code was developed using a computer random number generator in SPSS to select random permuted blocks (specified allocation ratio 1:1). The block lengths were 4 or 6, depending on the number of YHC teams that participate per MHS. Within the MHSs an even number of YHC teams were randomly allocated to the two study arms: an intervention and a control group. The teams in the intervention group offer the prevention protocol to parents of overweight children, and in the control group the teams offer usual care to these parents. The effects of the prevention protocol will be evaluated after two years of follow-up by comparing the outcomes of BMI and waist circumference of the overweight children with those of the children in the control group, taking into account the baseline values of these measures [20,27]. Data collection started in September 2007 and will continue until August 2010. The Medical Ethics Committee of the Erasmus Medical Centre Rotterdam approved the study protocol (reference number MEC-2007-163).

### Study procedure

A few weeks before the regular preventive health check is scheduled, all parents receive information about the study 'Be active, eat right' at home by mail and are invited to provide written informed consent for participation in the study. In addition, all parents are invited to complete a two-page questionnaire to measure data on demographic factors, overweight inducing/reducing behaviours (i.e. being physically active, having breakfast, drinking sweet beverages and watching television/playing computer games), their attitudes regarding these behaviours, and the health-related quality of life of their children. With this information a non-response analysis can be performed.

During the preventive health check, the YHC teams register the measures of weight, height and waist circumference of the children, calculate the BMI, and classify all children as normal weight, overweight or obese according to the international age and gender specific cut-off points of BMI [27]. In the control group whenever a YHC team detects a child with overweight, they apply usual care. In general, this implies giving basic information to the parents during the regular preventive health check about the importance of good nutrition and physical activity.

In the intervention group, the subgroup of parents of overweight children are offered up to three additional structured lifestyle counselling sessions, according to the prevention protocol. During these sessions the focus is on four behaviours, i.e. being physically active, having breakfast, drinking sweet beverages, and watching television/playing computer games [28]. These particular behaviours were chosen based on a literature review reporting on the most promising elements to prevent overweight [25]. During the counselling sessions, parents receive personal advice about a healthy lifestyle and are motivated for and assisted in behavioural change.

At the end of the regular preventive health check, the subgroup of parents with overweight children (in both groups) are invited to complete an additional questionnaire. This questionnaire provides more specific data about the baseline levels of overweight inducing/reducing behaviours, attitudes of parents regarding these behaviours, and the health-related quality of life of the children.

### Participants

#### Municipal Health Services and Youth Health Care teams

The managers of the MHSs, managers of the YHC department, and managers of the department of health education of all 37 MHSs in the Netherlands were informed about the study by mail and were contacted by the researchers by telephone in the first half of 2007. From the 37 MHSs, 9 volunteered to participate in the study. Of the remaining MHSs, 3 did not meet the inclusion criteria (i.e. MHSs should have YHC teams that had not used the prevention protocol before), 25 MHSs had other reasons not to participate (e.g. a recent or upcoming merger of MHSs). Of the 9 participating MHSs, a total of 44 YHC teams were willing to participate in the study. When a professional worked in more than one YHC team, the team that invited the most children for the health check during the school year 2007/2008 was selected for participation, and the other team was excluded from participation. At the start of the study no major changes were expected in the composition of the participating teams. The participating teams cover both urban and rural regions in the Netherlands. Prior to the start of the study, the research group arranged meetings to explain the procedure of the study and to instruct the participating YHC professionals.

#### Children and their parents

**Figure 1:**
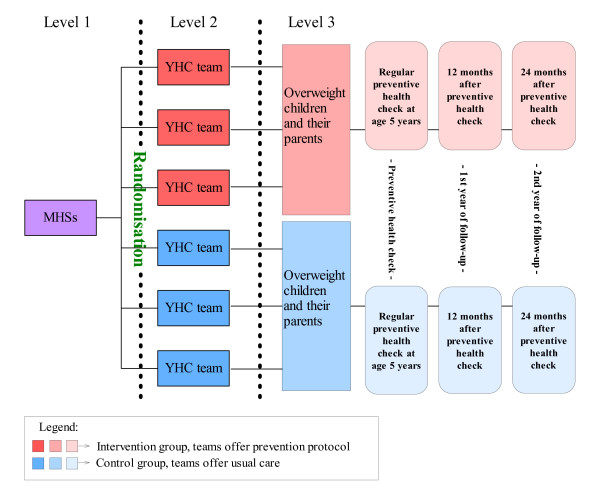
**Flow chart of the design of the study**.

**Figure 2:**
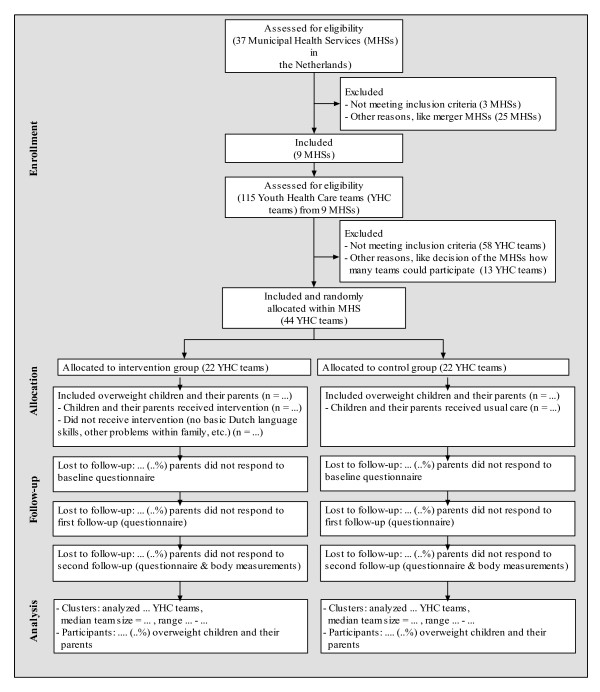
**Flow of the clusters and participants through the trial**.

The study population consists of the subgroup of children with overweight according to the international age and gender specific cut-off points for BMI. Parents and children will be excluded from analysis if the children have chronic health problems that may influence the outcome measures. In order to participate the parents should have at least basic Dutch language skills. The study design and participant flow are shown in Figures 1 and 2.

### Intervention

The prevention protocol (see appendix) is based on theories and models of behavioural change, i.e. the ASE model, a theoretical model of exercise habit formation, the Precaution Adoption Process Model, the Elaboration Likelihood Model, the stages of change model, and motivational interviewing techniques [29-34]. During the regular preventive health check, when a child in the intervention group with overweight is detected, the parents are offered up to three additional structured lifestyle counselling sessions to promote overweight-preventing behaviours. Prior to the start of the study, the YHC professionals in the intervention group received training in a non-directing guiding style as part of the prevention protocol [35]. The YHC professionals assess whether the parents are motivated to participate in this counselling, and will make use of a motivational interview approach if needed [35]. The three additional structured lifestyle counselling sessions are offered to parents with intervals of 1, 3 and 6 months after the regular preventive health check. The content of each visit depends on the stage of behavioural change that the parents are in [33]. The purpose of the sessions is to make parents aware of the overweight of their child, to provide information about overweight and its consequences, and to motivate the parents for and assist them in behavioural change. Materials that are used during the sessions are: a form for the YHC professional to assess the behaviours that should be targeted within the family, and diaries on energy intake and expenditure to be completed by the parents. Table 1 shows the guidelines for the four target behaviours for children at the age of about 5 years. The YHC professional and the parents together draw up a family-oriented action plan aiming at the promotion of physical activity and outdoor playing time, having family breakfast daily, consuming less sweet drinks and/or limiting watching television/playing computer games (Table 2) [25]. A pilot study has established the feasibility and acceptability of the prevention protocol [36].

**Table 1:** Guidelines used during counselling sessions regarding the four target behaviours for children aged 5 years.

**Behaviour**	**Guideline**
Being physical active	- At least 1 hour each day
	- Moderate intensity (outdoor playing, walking, cycling or doing sport)
Having breakfast	- Daily
	- In the family setting
Drinking sweet beverages	- Not more than 2 glasses per day (of soft drinks, fruit juices, sports/energy drinks, sweetened milk/yoghurt drinks or tea with sugar)
Watching television/playing computer games	- Not more than 2 hours per day (watching television and playing computer games combined)

### Measurements

#### Primary outcomes

##### Body measurements

Standardised methods are used to measure weight, height and waist circumference of all children. The YHC professionals received training in measuring the waist circumference of the children and all use the same type of measuring tape (SECA 200) provided by the researchers. BMI is calculated using weight in kilogram divided by squared height in metres. The YHC professionals received a calculator with instructions on how to calculate BMI. At baseline the YHC professionals classify the children into groups of normal weight, overweight or obese, according to the age and gender-specific cut-off points for BMI as published by the International Obesity Task Force (IOTF) [27,37]. After two years of follow-up the anthropometric measures will be repeated.

#### Secondary outcomes

##### Four target behaviours

In the questionnaire (2 pages) and the additional questionnaire booklet (including questions from SQUASH [38], CHQ-PF28 [39] and SDQ [40]) parents report (for weekdays and weekend days) the following:

- the frequency and duration of physical activity and outdoor playing time of their children

- how often their children have breakfast

- the intake of sweet beverages of their children

- the frequency and duration of inactivity of their children due to watching television and/or playing computer games

Data on parenting styles, parenting practices and attitude of the parents concerning the four target behaviours are assessed. Examples are: behaviour of the parents themselves, family rules about watching television/playing computer games, and availability at home of sweet beverages and breakfast products. After 12 and after 24 months of follow-up a questionnaire to assess this data will be repeated.

Other characteristics that will be taken into account include:

- demographics: gender, ethnicity of the children and parents, educational level of the parents, household and family composition, and neighbourhood characteristics (i.e. can children play safely outside; presence of busy roads, etc.)

- self-reported weight and height of the parents themselves

- participation in weight-management programs other than those used in the present study

- general health of the children (measured with the 28-item Child Health Questionnaire (CHQ-PF28; [39])

- health-related quality of life, and emotional/behavioural problems of the children [40]

- indicators of negative side effects (i.e. worry, stigmatization and lower self-esteem related to the weight of the children, and development of relative underweight [20])

### Sample size

Sample size was calculated taking into account the intra-cluster correlation coefficient (ρ = 0.1), the number of clusters (44), the expected prevalence of overweight children in the study population, the standard deviation (SD), expected effect (a difference in mean), and the power of the study (80%). With a participation of 50%, an expected prevalence of overweight children of 9% and a loss-to-follow-up of 30%, at least 1,1301 children (and their parents) should be invited by the YHC teams to participate in the study to have a final sample of about 356 overweight children (178 in both the intervention and control group). Assuming a SD of BMI to be 1.0 kg/m^2 ^[36], a difference in mean BMI of 0.35 kg/m^2 ^between the children in the intervention group and the children in the control group can be established under the assumptions mentioned above. Assuming an SD of the average number of hours per day of watching TV, video, DVD and playing computer games combined to be 60 minutes per day [36,41,42], a difference of 20 minutes per day can be established.

### Statistical analysis

The aim of the study is to assess the effectiveness of the prevention protocol among children with overweight. An intention-to-treat analysis will be applied [43]. Multi-level analyses will be applied because of the three-level structure of the study, i.e. correlation of the repeated observations within a participant and the correlation of the observations of participants within a YHC team [26,44]. Linear multilevel analysis will be applied for continuous outcome variables and logistic multilevel analysis for dichotomous outcome variables [44]. Biometric and behavioural outcomes of the children at age 7 years will be analysed with independent variables: intervention or control group, gender, age, socio-economic status, ethnicity, weight of the parents, and baseline levels of the outcome variables. Interaction effects of gender, social disadvantage and ethnic background with the effect of the prevention protocol will be explored.

### Process evaluation: non-response, adherence and cost-effectiveness

In addition to the effect evaluation a process evaluation will be carried out.

A non-response analysis will be conducted to determine possible selection bias. In the non-response analysis the following characteristics of (non)-participating children and their parents will be considered: ethnicity of the parents and children, educational level of the parents, household composition, an indication of the levels of the four target behaviours reported by the parents, and self-reported BMI of the parents and their children. For adherence to the prevention protocol the following variables are registered: classification of the children by the YHC professionals to the correct weight status according to the international age and gender specific cut-off points of BMI, the number of sessions the parents of overweight children attend, and the intensity of the sessions (i.e. did the parents complete energy intake and/or expenditure diaries, draw up a family-oriented action plan with the YHC professional, etc.). Adherence of both the YHC professionals and parents to the different elements of the prevention protocol will be analysed in relation to changes in BMI, waist circumference, and lifestyle of the children by multiple linear or logistic regression analysis (depending on the type of outcome variable). Analysis of these variables may indicate which elements of the prevention protocol work (or do not work), and for whom. In addition, satisfaction with the protocol of parents and YHC professionals will also be assessed. Finally, a cost-effectiveness analysis will be performed using a societal perspective, including program and parents costs.

## Discussion

This study presents the design of a cluster randomised controlled trial on the prevention of overweight and obesity in children. The study evaluates a protocol that is proposed for application in the YHC setting for the prevention of overweight and obesity in children.

It is hypothesised that, after two years of follow-up, overweight children in the intervention group will have less BMI and waist circumference, spend more time being physically active, more frequently have family breakfast on a daily basis, consume less sweet beverages, and spend less time watching television/playing computer games compared to overweight children in the control group. Differences between subgroups (ethnicity and socio-economic status) regarding the effects of the prevention protocol, and determinants of overweight and obesity, will be described. Several process variables will be registered to measure whether differences exist in subgroups of adherence to the prevention protocol, concerning the positive effects on BMI, waist circumference and lifestyle. This will also provide insight into the effective elements of the prevention protocol.

Strengths of the study are the size of the study (44 YHC teams), the random controlled design, and the regular preventive health check of the MHSs which more than 95% of all invited parents and their children attend [45]. Children receive a YHC check-up at set ages, which offers optimal opportunity to provide tailored prevention. The follow-up at 12 and 24 months allow to investigate the long-term effects of the prevention protocol. Regarding the generalisability of the study results, a first strength is that it is a controlled study conducted in the practice setting. The intervention is applicable in the daily practice of the YHC professionals, which will facilitate implementation of the prevention protocol if it is found to be effective. A second strength regarding generalisability is that the participating YHC teams cover both urban and rural areas. A limitation of the study is that the behaviour of the children and their parents is based on self-reports by the parents.

In conclusion, this study evaluates a protocol for the prevention of overweight and obesity in children. The results of this study will provide insight into the effectiveness of the prevention protocol used in Youth Health Care, and in the determinants of overweight and obesity of children aged 5 to 7 years.

## Competing interests

All authors (L Veldhuis, MK Struijk, W Kroeze, A Oenema, CM Renders, AMW Bulk-Bunschoten, RA HiraSing and H Raat) declare that they have no competing interests.

## Authors' contributions

HR and RH had the original idea for the study and its design, and were responsible for acquiring the study grant. LV further developed the study protocol and is responsible for the data collection, data analysis and reporting the study results. WK helps to coordinate the study, and helped in developing intervention instruments and questionnaires. CR and AB help to coordinate the study. MS helps to coordinate the study and participates in data collection. CR, AB and AO provide expert input during the study. HR and RH supervise the study. All authors regularly participated in discussing the design and protocols used in the study. All authors read and approved the final manuscript.

## Appendix

### Description of the intervention: the different elements of the prevention protocol

- The YHC professional classifies children as normal weight, overweight or obese during the regular preventive health check

- The YHC professional offers parents of overweight children up to three additional lifestyle counselling sessions

- The YHC professional assesses whether the parents are motivated to participate

- The YHC professional will use the motivational interview approach if necessary

- The YHC professional assesses the behaviour(s) that should be targeted

- The YHC professional gives health-promoting and personal advice to the parents

- The YHC professional motivates parents for behavioural change

- The YHC professional and parents together draw up an action plan

- Parents complete diaries on energy intake and expenditure

- Intervals of the counselling sessions: 1 month, 3 months and 6 months

## Pre-publication history

The pre-publication history for this paper can be accessed here:


